# Health Complaints Associated With Poor Rental Housing Conditions in Arkansas: The Only State Without a Landlord's Implied Warranty of Habitability

**DOI:** 10.3389/fpubh.2018.00180

**Published:** 2018-06-19

**Authors:** Nathaniel Horwitz-Willis

**Affiliations:** ^1^Public Health Department, Plymouth, MA, United States; ^2^Massachusetts College of Pharmacy and Health Sciences, Boston, MA, United States

**Keywords:** URLTA, habitability, substandard housing, landlord and tenant, housing and health

## Abstract

This is a review of an existing article that surveyed the perceived health of renters' in Arkansas. As a first in the field of public health it was able to provide ground-level insight through tenant interviews about housing and health in the state. This review illuminates how the state's sociopolitical characteristics may affect marginalized Arkansas renters. Marginalized renters may include persons living below the poverty line, people of color, and women who are the head of household. This article seeks to elucidate how the upstream factor, habitability law, may impact tenant health in the state. With a novel public health approach, the article contributes to the existing housing and health literature with its invaluable insight of stakeholders' perspectives relating to how habitability law may impact their health outcomes. Now is an opportune time for public health practitioners, researchers, and policymakers to facilitate incremental change to include landlords in their state public health system. Such inclusion may improve housing while promoting, improving, and protecting health outcomes for Arkansas renters.

A systematic assessment undertaken by Bachelder et al. (1) elucidate how landlords may impact the health of tenants. The authors' description and analysis of renter perceived health-related problems corresponds to a lack of landlord housing repairs aligns with the latest housing and health literature, which details the associations between housing and health conditions (2–4) and most recently, how habitability policies may influence tenant health across the United States (5, 6). This reviewed article illuminates how the state's sociopolitical characteristics may affect an overlooked stakeholder—marginalized Arkansas renters. The major findings within the reviewed article illustrate that most renters were persons of color, compared to persons identified as white, were unaware that the state did not have tenant housing and health protections. Overall, the findings confer that the tenants' reported health problems can likely be ameliorated in the areas of general health, respiratory health, and mental health with improvements that focused on warmth and energy efficiency, ventilation improvement and high humidity avoidance previously identified in the literature (7). The linkages between housing and health are well-known (8, 9) and, in the face of compelling scientific evidence, Arkansas has yet to adopt an implied warranty of habitability that may enable landlords to be a critical component in changing health outcomes. Based on two previous articles (5, 6) related to the scope of this reviewed article, habitability is defined as: “Basic services, including heat, hot water, plumbing and a sound structure absent of physical defects not caused by the tenant, that do not pose unreasonable safety risks to the occupant residing in the housing unit.”

For landlords to be involved, legislators must develop policy that recognizes and welcomes the public health benefits of an implied warranty of habitability inclusive of landlord participation. For instance, the state of Massachusetts's housing code (10) requires Public Health Departments or their Boards of Health to enforce and act as the regulatory agency of such law governing tenant health, safety prevention, and protection. This legislation enables a landlord to become more closely involved in the public health system. Research indicates, housing policies and involving landlords can improve housing conditions to prevent or reduce the burden of chronic (11) and communicable diseases (12) in addition to behavioral or cognitive health problems (13). Based on the interviews conducted in the Bachelder et al. (1) study, it is apparent that future legislative direction would be to focus on crafting an implied warranty of habitability in Arkansas. A just and equal warranty of habitability, with requirements for both landlords and tenants, is an influential systemic upstream social determinant of health for renters.

The importance of implementing an implied warranty of habitability legal standard in Arkansas should not be taken lightly. The lack of such equitable regulation may stem from a state's geopolitical characteristics and should be further explored. Earlier findings indicate that states (*n* = 15) with characteristics similar to Arkansas that place all or a majority of the burden of a habitability standard upon tenants generally have majority Republican legislative bodies and weak or no standards in their law to hold the landlord accountable (5). The empirical research exploring such habitability policies also suggests inequitable habitability laws are likely found where a greater percent of marginalized groups of people rent. Marginalized groups of people may include persons living below the poverty line, people of color, and women that are the head of household. The referenced article, Bachelder et al. (1) used initial surveys to look at demographic characteristics (*N* = 1,108) and a marginalized group is indicated. It seemed that most renters surveyed (*n* = 951) who identified as persons of color (71%), compared to persons identified as white (11%), were unaware of the absence of housing and health protections in the state. It also appears that inadequate housing conditions may affect a considerable number of renters' chronic and acute health status who have incomes below $30,000 (65%), below the age of 54 (68%), educational attainment up to an Associate degree (89%), female (62%), and persons of color (87%). However, gathering more information from respondents' interviews (*n* = 5) to further understand potential health impacts from housing proved challenging. Despite the challenge, this paper is the first to identify literature for public health researchers to begin to understand how habitability policy might impact renters' health (5, 6).

The Uniform Residential Landlord Tenant Act (URLTA), is a model legislation designed to facilitate an opportunity for equitable and fair housing that may benefit the landlord and tenant if implemented at the state level and enforced within localities (6). There are many potential benefits that extend to both parties regarding the implementation and enforcement of such legislation for the landlord and tenant. Examples may include boosts to the local economy due to housing stock repair (14), opportunity for improved relations between landlord and tenant, and a potential higher likelihood that a tenant will not encounter derisions or weathering of their health and well-being associated with substandard housing issues out of their control (15).

In summary, future studies may consider replicating this research in similar settings as this study demonstrates several housing and health implications for a marginalized population. Although the Bachelder et al. (1) study does not measure health status it does demonstrate how housing may negatively impact one's perceived health status. Unfortunately, in Arkansas, a legal standard currently does not exist that enables enforcement of a habitability regulation from a public health agency that protects the health of tenants. The lack of this regulatory framework may contribute to, as the authors may have uncovered, a silent ongoing epidemic and amongst a “hidden” vulnerable class. As the research base continues to make connections between landlord-tenant law, housing, and tenant health this “hidden” and often marginalized population segment in Arkansas might hopefully benefit sooner rather than later, in the twenty-first Century, from an equitable landlord-tenant implied warranty of habitability standard. This reviewed article presents conclusive evidence to show, through perceived health status, marginalized populations' health generally is at-risk as a renter and how the absence of a tenant habitability law can be a method used to not afford them health protection depending on a state's geopolitical characteristics.

## Author contributions

The author confirms being the sole contributor of this work and approved it for publication.

### Conflict of interest statement

The author declares that the research was conducted in the absence of any commercial or financial relationships that could be construed as a potential conflict of interest.
